# Association of thyroid autoimmunity and pregnancy outcomes in unexplained recurrent pregnancy loss women: a prospective cohort study

**DOI:** 10.3389/fendo.2025.1711369

**Published:** 2025-11-25

**Authors:** Ruifang Wang, Ling Liu, Wei Zhang, Jian Zhang, Kexin Wang, Fang Wang

**Affiliations:** 1 Department of Reproductive Medicine, Lanzhou University Second Hospital, Lanzhou, China; 2 Department of Obstetrics and Gynecology, The First Affiliated Hospital, and College of Clinical Medicine of Henan University of Science and Technology, Luoyang, China

**Keywords:** thyroid autoimmunity, unexplained recurrent pregnancy loss, fertility, subsequent pregnancy outcome, pregnancy complications

## Abstract

**Background:**

Unexplained recurrent pregnancy loss (URPL) and thyroid autoimmunity (TAI) have received considerable attention. However, the association between TAI and subsequent infertility or pregnancy outcomes among euthyroid women with URPL remains unclear. This study aimed to clarify these relationships.

**Methods:**

From September 2019 to December 2022, we prospectively enrolled women with URPL at the Lanzhou University Second Hospital and collected pre-pregnancy and first-trimester data. Participants were divided into two groups: TAI and non-TAI, based on their thyroid antibody status. Changes in thyroid function parameters from pre-pregnancy to the first trimester were compared between the two groups. We assessed the incidence of infertility, subsequent pregnancy loss, and pregnancy complications. Logistic regression analysis was performed to examine the associations between TAI and infertility, subsequent pregnancy loss, and adverse pregnancy outcomes, after adjusting for maternal age, body mass index, gravidity, number of pregnancy losses, and concentrations of thyroid-stimulating hormone (TSH) and free thyroxine (fT4).

**Results:**

Of the 576 euthyroid women with URPL included, 101 (17.5%) were classified as TAI, and 475 (82.5%) as non-TAI. Subsequently, 110 (19.1%) patients were diagnosed with infertility and 466 (80.9%) patients conceived. The mean TSH concentrations in the TAI group were higher than those in the non-TAI group both pre-pregnancy (2.3 ± 0.8 vs. 2.2 ± 0.9 mIU/L, P = 0.085) and in the first trimester (2.1 ± 1.2 vs. 1.7 ± 0.9 mIU/L, P = 0.014). The mean fT4 concentration in the first trimester was lower in the TAI group (14.2 ± 2.8 vs. 14.9 ± 1.9 ng/ml, P = 0.024). Logistic regression analysis showed that TAI was independently associated with increased risks of subsequent pregnancy loss (OR 2.953, 95%CI 1.142–3.693), hypothyroidism during pregnancy (OR 5.567, 95%CI 3.035–10.210), premature rupture of membranes (OR 2.198, 95%CI 1.051–4.595), and preterm birth (OR 2.865, 95%CI 1.132–7.249).

**Conclusions:**

Among euthyroid women with URPL, TAI is independently associated with increased risks of subsequent pregnancy loss, hypothyroidism during pregnancy, premature rupture of membranes, and preterm birth, underscoring the need for pre-pregnancy attention.

## 1 Introduction

Recurrent pregnancy loss (RPL) is defined as the spontaneous loss of two or more clinical pregnancies before the 24th week of gestation, excluding ectopic and molar pregnancies, confirmed by ultrasonography or histopathologic examination (1). RPL affects up to 3% of couples of reproductive age (1, 2). The etiology of RPL is complex; maternal age and the interval since the previous miscarriage are considered main risk factors. Other contributing factors include chromosomal or genetic abnormalities, anatomical defects, autoimmune diseases, prothrombotic conditions, endocrine factors, infections, male-related factors, and environmental or psychological stressors (1–5). Nevertheless, more than 50% of RPL cases remain unexplained and are therefore classified as unexplained recurrent pregnancy loss (URPL) (1, 2, 6). Previous research has indicated that URPL is associated with immune abnormalities (7, 8).

Thyroid autoimmunity (TAI) is defined as the presence of thyroid peroxidase antibodies (TPO-Ab) or thyroglobulin antibodies (TG-Ab), regardless of thyroid function (9, 10). The prevalence of TAI ranges from 5% to 15% among women of childbearing age (11), and is even higher in pregnant women, with reported rates between 5% and 25% (12–17). TAI is associated with increased risks of miscarriage, preterm birth, and subfertility, although the underlying mechanisms remain poorly understood (10, 18–23). Previous evidence indicates that women with RPL have a higher prevalence of TAI, ranging from 16% to 36% (16, 24–26). However, whether TAI contributes to URPL and influences subsequent pregnancy outcomes remains controversial.

Prospective cohort studies and meta-analyses have reported inconsistent findings regarding the association between TAI, RPL, and obstetrical complications (22, 27–29). Recently, an increasing number of studies have focused on the impact of TAI on fertility and pregnancy outcomes (16–18, 23, 29), and have suggested that thyroid hormone replacement does not improve pregnancy outcomes (30, 31). Thus, the relationship between TAI and these outcomes remains unclear, making it difficult to draw definitive conclusions. Furthermore, TAI may contribute to adverse pregnancy outcomes not only through thyroid destruction, but also via other mechanisms. One possible explanation for this uncertainty is that many studies had small sample sizes, focused on a single outcome, and did not account for confounding variables.

Accordingly, using our pregnancy loss cohort, we aimed to examine the association of TAI on fertility, pregnancy outcomes, and pregnancy complications in euthyroid women with URPL.

## 2 Materials and methods

### 2.1 Study design and subjects

This prospective cohort study included 2,493 women with a history of at least one pregnancy loss who visited the Reproductive Center of Lanzhou University Second Hospital (Lanzhou, China) between September 2019 and December 2022. Details of the cohort design have been described in our previous publication (32). RPL was defined as two or more pregnancy losses prior to 24 weeks of gestation with the same partner. URPL was defined as RPL with no identifiable cause. All patients underwent a standardized diagnostic protocol, and those diagnosed with URPL were enrolled in this study.

We collected comprehensive patient information, including obstetric history, age, education, ethnicity, age at menarche, age at first pregnancy, body mass index (BMI), number and type of previous pregnancy losses, and the final outcome of the current pregnancy. In addition, serum levels of thyroid-stimulating hormone (TSH), free triiodothyronine (fT3), and free thyroxine (fT4) were measured. All patients underwent TPO-Ab and TG-Ab tests. Patients who tested positive for TPO-Ab and/or TG-Ab were assigned to the TAI group, while those who tested negative for both antibodies were assigned to the non-TAI group. Furthermore, both groups were required to meet the following inclusion and exclusion criteria.

Patient inclusion criteria were as follows (1): age between 18 and 42 years (2); history of two or more pregnancy losses (3); non-pregnant at the time of the initial visit (4); complete baseline data and laboratory evaluations (5); expressed a desire to conceive again (6); at least one year of follow-up; and (7) euthyroid function (normal levels of TSH and free thyroxine).

Exclusion criteria included (1): known high-risk factors for pregnancy loss, including chromosomal abnormalities (embryonic or parental), abnormal anatomical structure (for example, uterine mediastinum), autoimmune diseases (such as connective tissue disease, systemic lupus erythematosus, rheumatoid arthritis, and Sjogren’s syndrome), prothrombotic state, endocrine factors (such as diabetes, hyperthyroidism, and hypothyroidism), infection factors (e.g., tuberculosis, rubella virus, syphilis, endometritis), or male factors (e.g., asthenozoospermia) (2); conception via assisted reproductive technologies; and (3) significant cardiovascular, hepatobiliary, renal, hematopoietic, or other medical conditions.

**Figure 1:**
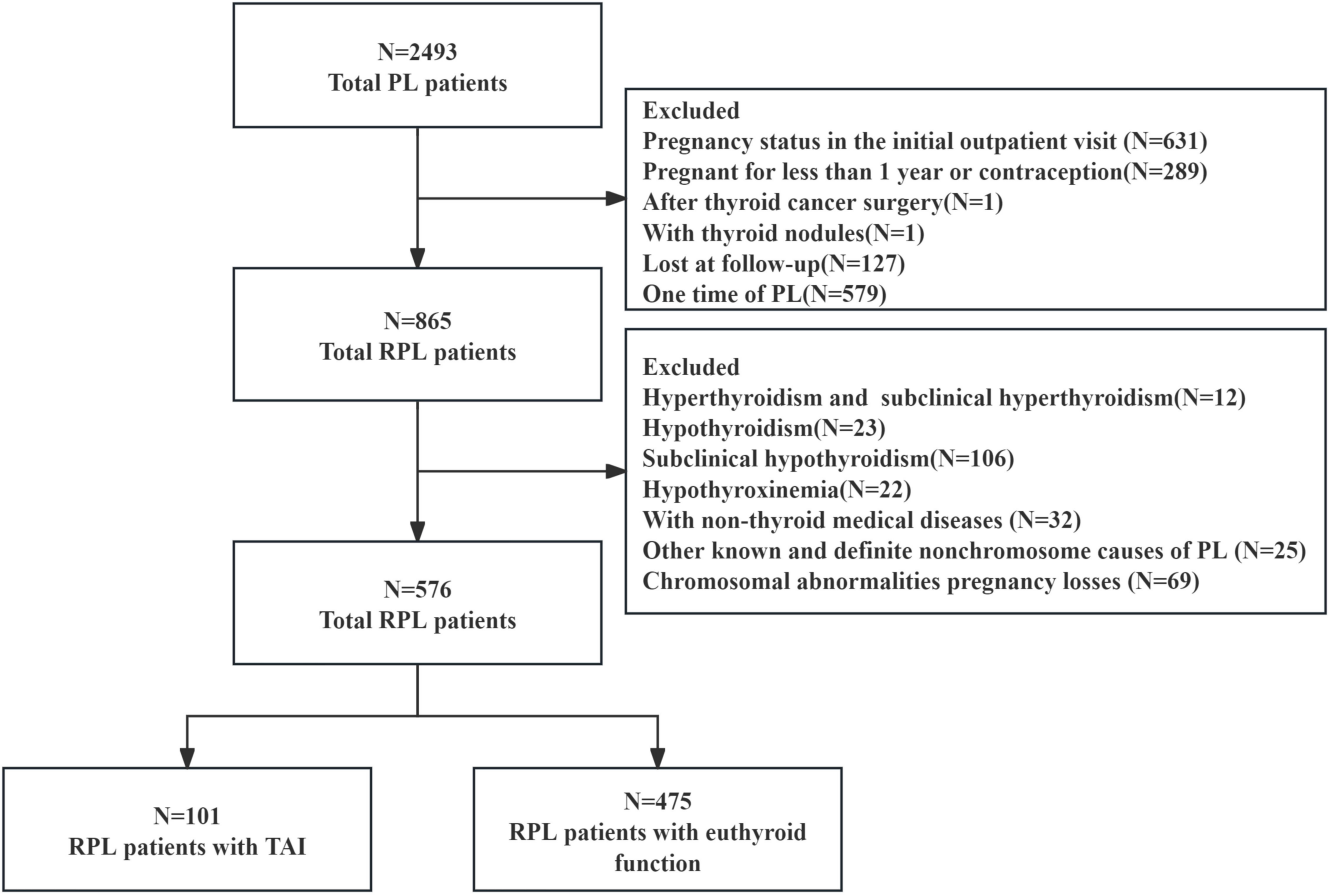
Flow chart of research population selection.

Additionally, 127 participants (5%) were lost to follow-up. Finally, 576 euthyroid, non-pregnant women with a history of URPL were enrolled (**Figure 1**).

### 2.2 Laboratory assessments and treatment

Blood samples were collected by venipuncture of the brachial vein at the Laboratory Medical Center of Lanzhou University Second Hospital. Detection was performed using an electrochemiluminescence immunoassay. Thyroid function parameters measured included triiodothyronine (T3), thyroxine (T4), TSH, fT3, fT4, TG-Ab, and TPO-Ab. Diagnostic criteria for TAI and euthyroidism were based on the reference ranges established by the Laboratory Medical Center of the Lanzhou University Second Hospital. The reference ranges were as follows: T3, 1.3–3.1 nmol/L; T4, 66–181 nmol/L; TSH, 0.270–4.200 mIU/L; fT4, 12.0–22.0 ng/mL; fT3, 3.10–6.80 pmol/L; TPO-Ab, 0–60 mIU/L; and TG-Ab, 0–60 mIU/L. The cut-off values for positivity were 60 mIU/L for both TPO-Ab and TG-Ab. TAI was defined as positivity for TPO-Ab and/or TG-Ab. Euthyroid function was defined as all serum thyroid function parameters within the normal reference range. Hypothyroidism in pregnancy was defined as TSH >4.0 mIU/L and fT4 <12 ng/mL, regardless of thyroid antibody status. Subclinical hypothyroidism in pregnancy was defined as TSH >2.5 mIU/L with normal fT4, in the first trimester, irrespective of thyroid antibody status (33). Hyperthyroidism in pregnancy was defined as TSH <0.27 mIU/L and fT4 >22 ng/mL, regardless of thyroid antibody status. Subclinical hyperthyroidism in pregnancy was defined as TSH <0.27 mIU/L with normal fT4 during the first trimester, regardless of thyroid antibody status.

If hypothyroidism or subclinical hypothyroidism was diagnosed in early pregnancy, levothyroxine (LT4) therapy was initiated promptly, and thyroid function was regularly monitored and maintained throughout pregnancy. Patients diagnosed with hyperthyroidism or subclinical hyperthyroidism were referred to the endocrinology department for further evaluation. Based on the evaluation, initiation of antithyroid medication (preferably Methimazole), regular monitoring of thyroid function, and timely discontinuation of therapy were determined as appropriate.

### 2.3 Clinical outcomes

Clinical outcome data were collected using the reproductive follow-up system of Lanzhou University Second Hospital. The sources of data included medical records (obtained from face-to-face outpatient visits and the hospital information system for inpatients), laboratory test results, as well as follow-up via WeChat and telephone.

Outcome data collected included infertility, normal fertility, pregnancy loss, live birth, and non-live birth. In this analysis, only the first pregnancy outcome after cohort entry was included. Infertility was defined as the inability to conceive after 12 months or more of regular, unprotected intercourse (34). Participants who did not achieve pregnancy within two years after cohort entry were not further followed. Normal fertility was defined as achieving a clinical pregnancy within 12 months of attempting conception. Pregnancy was defined as a positive urine or serum β-hCG test or the confirmation of a gestational sac by ultrasound. Pregnancy loss was defined as a spontaneous loss before 24 weeks of gestation. Live birth was defined as fetal survival beyond 28 weeks of gestation, according to Chinese criteria. Non-live birth included pregnancy loss, molar pregnancy, ectopic pregnancy, and stillbirth. From medical records, information was collected on adverse pregnancy complications, including gestational diabetes mellitus (GDM), anemia during pregnancy, hypothyroidism in pregnancy, pregnancy-induced hypertension, intrahepatic cholestasis of pregnancy, fetal distress *in utero*, fetal growth restriction (FGR), oligohydramnios, premature rupture of membranes (PROM), preterm delivery, and postpartum hemorrhage. Neonatal outcomes collected included birthweight, Apgar score, need for intensive neonatal care, and neonatal mortality.

Adverse pregnancy complications included both maternal and fetal-neonatal complications, with definitions based on standard clinical guidelines for each condition. Gestational diabetes mellitus (GDM) was diagnosed using a 75-g oral glucose tolerance test (OGTT), according to the 2010 guidelines of the International Association of Diabetes and Pregnancy Study Groups (35). If one or more plasma glucose (PG) values met or exceeded the set thresholds (fasting PG 5.1 mmol/L, 1h-PG 10.0 mmol/L, 2h-PG 8.5 mmol/L), Women with overt diabetes diagnosed before pregnancy were excluded. Anemia in pregnancy was defined as a hemoglobin (Hb) concentration <110 g/L (36). Hypothyroidism in pregnancy included hypothyroidism and subclinical hypothyroidism diagnosed in the first trimester. Pregnancy-induced hypertension (PIH) was defined as new-onset hypertension (blood pressure ≥140/90 mmHg) developing after 20 weeks’ gestation, with or without proteinuria or edema (37). Intrahepatic cholestasis of pregnancy was defined by the presence of pruritus and elevated serum bile acid levels (38). Fetal distress *in utero* was defined as evidence of fetal hypoxia or malnutrition during pregnancy. FGR was defined as fetal growth below the 10th percentile for gestational age, based on the Growth Standard Curves for Chinese Newborns established by the Capital Institute of Pediatrics (39). Oligohydramnios was defined as amniotic fluid volume less than the 5th percentile for gestational age, an amniotic fluid index <5 cm, or a single deepest pocket <2 cm. PROM was defined as rupture of the amniotic sac before the onset of labor (40). Preterm birth was defined as delivery occurring between 28 and less than 37 weeks of gestation. Postpartum hemorrhage was defined as a cumulative blood loss of ≥500 mL following vaginal delivery or ≥1,000 mL following cesarean delivery.

### 2.4 Statistical analysis

Data were presented as mean ± standard deviation (SD) for continuous variables, and as counts and percentages for categorical variables. Differences between two groups were assessed using independent samples t-tests, while differences among three or more groups were analyzed by one-way analysis of variance (ANOVA). For categorical variables, the two-sided Pearson chi-square test or Fisher’s exact test was used, as appropriate. Pearson correlation analysis was also performed where applicable. The Bonferroni correction was applied for multiple group comparisons. Variables with P < 0.1 in univariate analyses were included in multivariate logistic regression models to calculate odds ratios (ORs) for the association between risk factors and infertility, non-live birth, and subsequent pregnancy loss. A P value < 0.05 was considered statistically significant. All statistical analyses were performed using SPSS for Windows, version 27.0 (SPSS Inc., Chicago, IL, USA).

One-way ANOVA or the Kruskal-Wallis test, with Bonferroni adjustment for pairwise comparisons, was used to compare continuous variables across groups. Univariate associations between continuous variables were assessed using the Spearman rank correlation coefficient. We used analysis of covariance (ANCOVA) on log-transformed birth-weight (and weight centile) to test whether the interaction between TPO-Ab positivity and TSH concentration influenced fetal growth. For categorical variables with small expected cell counts (<5), the chi-squared test or Fisher’s exact test was used as appropriate. To compare categories in multi-way contingency tables, chi-squared statistics were partitioned using Bonferroni correction. The relationships between exposure variables, potential confounders (nulliparity, first trimester TSH levels, maternal age, first trimester BMI, antinuclear antibody positivity, or rheumatic diseases), and pregnancy outcomes were assessed using penalized logistic regression analysis. Model fit was assessed using the Hosmer-Lemeshow test and pseudo R^2^. The Box-Tidwell test was used to verify the linear relationship between log odds and continuous independent variables (STATA 13.0, College Station, TX 2013). To account for confounders, logistic equations were also utilized to test for interactions between variables. *A priori* sample size calculation was not performed, as this was not a randomized study.

## 3 Results

### 3.1 Baseline characteristics

In this study, 576 euthyroid women with URPL were involved. Among them, 475 (82.5%) were classified as non-TAI and 101 (17.5%) as TAI. Within the TAI group, 42 women (41.6%) were positive for TPO-Ab only, 30 (29.7%) were positive for TG-Ab only, and 29 (28.7%) were positive for both antibodies. In the TAI group, the mean TSH concentration measured before pregnancy was higher than that in the non-TAI group (2.4 ± 0.9 mIU/L vs. 2.2 ± 0.8 mIU/L, P = 0.037). The mean fT3 concentration was lower in the TAI group (5.1 ± 0.4 pmol/L vs. 5.2 ± 0.5 pmol/L, P = 0.018). There were no statistically significant differences in the mean concentrations of T3, T4, or fT4 between the two groups (**Table 1**).

**Table 1:** Baseline characteristics of 576 euthyroid women with URPL.

Variables	Whole patients (N = 576)	TAI group (N = 101)	Non-TAI group (N = 475)	*P*
Maternal Age (years)	30.3 ± 4.0	30.9 ± 3.7	30.2 ± 4.1	0.110
BMI (kg/m^2^)	22.4 ± 3.1	23.0 ± 3.5	22.2 ± 3.0	**0.017**
BMI (kg/m^2^)				
<18.5	47(8.2)	6(5.9)	41(8.6)	0.080
18.5-23.9	393(68.2)	63(62.4)	330(69.5)	
24-27.9	101(17.5)	21(20.8)	80(16.8)	
≥28(obesity)	35(6.1)	11(10.9)	24(5.1)	
Ethic				
Han	515(89.4)	84(83.2)	431(90.7)	**0.025**
Other	61(10.6)	17(16.8)	44(9.3)	
Education				
Junior middle school and below	102(17.7)	10(9.9)	92(19.4)	**0.017**
High school and junior college	241(41.8)	39(38.6)	202(42.5)	
Bachelor’s degree or above	233(40.5)	52(51.5)	181(38.1)	
Age at menarche (years)	13.5 ± 1.2	13.6 ± 1.3	13.4 ± 1.2	0.130
Age at first pregnancy (years)	26.0 ± 3.4	26.6 ± 3.4	25.9 ± 3.4	**0.038**
Number of total pregnancy	2.9 ± 1.1	2.9 ± 0.9	2.9 ± 1.1	0.976
Number of pregnancy loss	2.5 ± 0.8	2.4 ± 0.8	2.5 ± 0.8	0.385
Type of pregnancy loss				
Primary	446(77.4)	72(71.3)	374(78.7)	0.104
Secondary	130(22.6)	29(28.7)	101(21.3)	
Thyroid function before pregnancy				
T3 (nmol/L)	1.8 ± 0.3	1.9 ± 0.3	1.8 ± 0.3	0.237
T4 (nmol/L)	111.2 ± 19.6	112.7 ± 19.1	110.8 ± 19.7	0.375
fT3 (pmol/L)	5.2 ± 0.5	5.1 ± 0.4	5.2 ± 0.5	**0.018**
fT4 (ng/ml)	15.9 ± 1.9	15.9 ± 1.9	15.9 ± 1.9	0.810
TSH (mIU/L)	2.2 ± 0.9	2.4 ± 0.9	2.2 ± 0.8	**0.037**
TSH (mIU/L)				
<2.5	368(63.9)	59(58.4)	309(65.1)	0.207
≥ 2.5 to ≤ 4.2	208(36.1)	42(41.6)	166(34.9)	

Continuous variables are described as mean and standard deviation, categorical variables are expressed as numbers and percentages. BMI, body mass index; T3, triiodothyronine; T4, thyroxine; fT3, free triiodothyronine; fT4, free thyroxine; TSH, thyroid stimulating hormone.

*P* values <0.05 were shown in bold.

The baseline characteristics of the participants are summarized in **Table 1**. There were no significant differences between the TAI and non-TAI groups in age, maternal age, total number of pregnancies, or the number and type of previous pregnancy losses. Compared with the non-TAI group, participants in the TAI group had a higher BMI (23.0 ± 3.5 kg/m² vs. 22.2 ± 3.0 kg/m², P = 0.017), were older at the time of their first pregnancy (26.6 ± 3.4 vs. 25.9 ± 3.4 years, P = 0.038), had a higher proportion of individuals from other ethnic groups (16.8% vs. 9.3%, P = 0.025), and were more likely to have attained a bachelor’s degree or higher (51.5% vs. 38.1%, P = 0.017).

### 3.2 Evolution of thyroid function during first-trimester pregnancy

During follow-up, 110 (19.1%) patients were diagnosed with infertility, whereas 466 (80.9%) patients conceived subsequently. Among those who became pregnant, 368 (81.1%) had a live birth, and 86 (18.1%) patients had a subsequent pregnancy loss.

To characterize changes in thyroid function from pre-pregnancy to the first trimester among women with and without TAI, we measured T3, T4, fT3, fT4, TSH, TG-Ab, and TPO-Ab levels in both periods for all pregnant participants. In the TAI group, the mean TSH concentrations measured in pre-pregnancy period and the first trimester were higher than those in the non-TAI group (pre-pregnancy: 2.3 ± 0.8 mIU/L vs. 2.2 ± 0.9 mIU/L, P = 0.085, and first trimester: 2.1 ± 1.2 mIU/L vs. 1.7 ± 0.9 mIU/L, P = 0.014). The mean concentrations of fT3 in both pre-pregnancy period and the first trimester were lower in the TAI group (pre-pregnancy: 5.1 ± 0.4 pmol/L vs. 5.3 ± 0.5 pmol/L, P = 0.038 and first trimester: 4.7 ± 0.7 pmol/L vs. 5.0 ± 0.5 pmol/L, P<0.001). In addition, the mean fT4 concentration in the first trimester was lower in the TAI group than in the non-TAI group (14.2 ± 2.8 ng/ml vs. 14.9 ± 1.9 ng/ml, P = 0.024). Furthermore, higher proportions of women in the TAI group had first-trimester TSH concentrations ranging from 2.5 to 4.0 mIU/L (18.7% vs. 10.7%) or exceeding 4.0 mIU/L (8.0% vs. 1.8%), compared with the non-TAI group (P = 0.001) (**Supplementary Table S1**).

In the TAI group, first-trimester TSH concentrations were not significantly different from pre-pregnancy levels (2.1 ± 1.2 mIU/L vs. 2.3 ± 0.8 mIU/L, P = 0.156), while in the non-TAI group, first-trimester TSH concentrations were significantly lower than pre-pregnancy levels (1.7 ± 0.9 mIU/L vs. 2.3 ± 0.9 mIU/L, P<0.001). However, both TAI and non-TAI women exhibited a decline in T4, fT4 and fT3 levels during early pregnancy (**Table 2**).

**Table 2:** Comparisons of thyroid function indexes between pre-pregnancy and the first trimester in 466 pregnant women.

	TAI group(N = 75)		*P_1_*	Non-TAI group(N = 391)		*P_2_*
	Pre-pregnancy	First-trimester		Pre-pregnancy	First-trimester	
T3 (nmol/L)	1.8 ± 0.3	1.7 ± 0.4	**0.018**	1.8 ± 0.3	1.8 ± 0.3	0.150
T4 (nmol/L)	112.8 ± 19.3	104.6 ± 23.1	**0.020**	111.4 ± 19.8	106.8 ± 17.8	**<0.001**
fT3 (pmol/L)	5.1 ± 0.4	4.7 ± 0.7	**<0.001**	5.3 ± 0.5	5.0 ± 0.5	**<0.001**
fT4 (ng/ml)	15.7 ± 1.9	14.2 ± 2.8	**<0.001**	16.0 ± 1.9	14.9 ± 1.9	**<0.001**
TSH (mIU/L)	2.3 ± 0.8	2.1 ± 1.2	0.156	2.2 ± 0.9	1.7 ± 0.9	**<0.001**
TPO-Ab (mIU/L)	340.1 ± 467.4	335.3 ± 448.0	0.948	28.7 ± 15.4	28.7 ± 18.5	0.992
TG-Ab (mIU/L)	109.9 ± 118.1	114.4 ± 102.0	0.761	18.1 ± 10.2	17.5 ± 13.8	0.505
TSH (mIU/L)						
<2.5	44(58.7)	55(73.3)	**0.019**	252(64.5)	342(87.5)	**0.000**
≥ 2.5 to ≤ 4.0	29(38.7)	14(18.7)		134(34.3)	42(10.7)	
>4.0	2(2.7)	6(8.0)		5(1.3)	7(1.8)	

Continuous variables are described as mean and standard deviation, categorical variables are expressed as numbers and percentages. T3, triiodothyronine; T4, thyroxine; fT3, free triiodothyronine; fT4, free thyroxine; TSH, thyroid stimulating hormone; TPO-Ab, thyroid peroxidase antibody; TG-Ab, antithyroglobulin antibody.

*P* values <0.05 were shown in bold.

*P_1,_*pre-pregnancy data vs first first-trimester data in TAI group; *P_2,_*pre-pregnancy data vs. first first-trimester data in non-TAI group.

When we analyzed the changes in thyroid function between TAI and non-TAI women who had live births or pregnancy loss, we observed similar patterns (**Supplementary Tables S2**, **S3**). Additionally, we compared changes in thyroid-function parameters from pre-pregnancy to the first trimester within the live-birth group and within the pregnancy-loss group (**Supplementary Table S4**).

### 3.3 Effects of TAI on clinical outcomes and pregnancy complications

**Figure 2:**
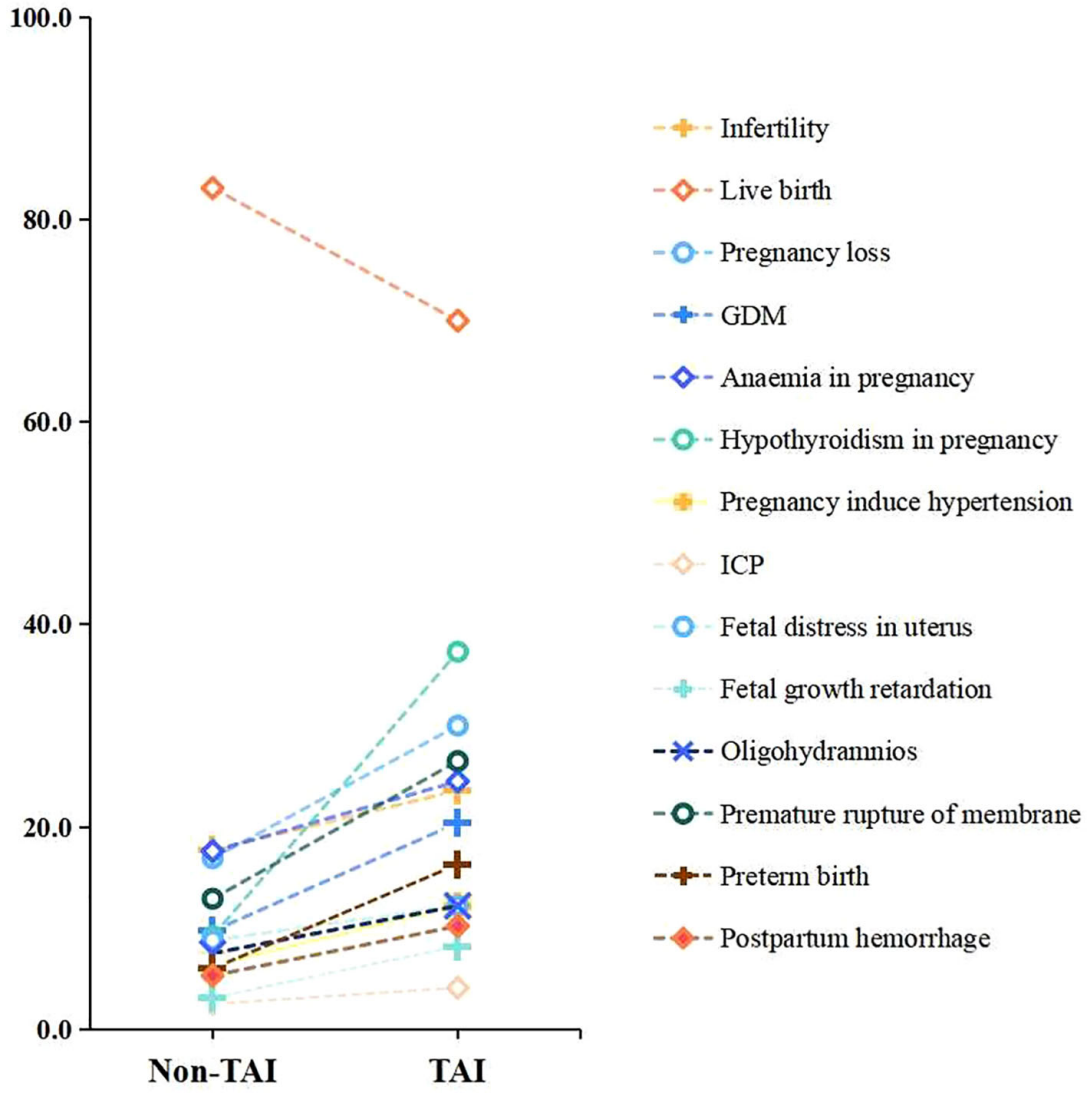
The prevalence of clinical outcomes and pregnancy complications were compared between the TAI and non-TAI groups.

**Figure 2** presents the prevalence of clinical outcomes and pregnancy complications in the TAI and non-TAI groups. Compared to the non-TAI group, women in the TAI group had a higher proportion of infertility, although the difference was not statistically significant (23.6% vs. 17.7%, P = 0.061) (**Table 3**). Women in the TAI group had significantly higher rates of subsequent pregnancy loss (30.0% vs. 16.9%, P = 0.010), GDM (20.4% vs. 9.7%, P = 0.027), hypothyroidism in pregnancy (37.3% vs. 9.2%, P<0.001), PROM (26.5% vs. 12.9%, P = 0.012), and preterm birth (16.3% vs. 6.0%, P = 0.017) (**Table 3**). Moreover, no significant difference was found in other pregnancy complications between the two groups.

**Table 3:** Proportions of infertility, subsequent pregnancy outcomes, adverse pregnant complications between groups.

Variables	Total	TAI group	Non-TAI group	*P*
Infertility				
Yes	110(19.1)	26(23.6)	84(17.7)	0.061
No	466(80.9)	75(74.3)	391(82.3)	
Subsequent pregnancy outcomes				
Live birth	368(81.1)	49(70.0)	319(83.1)	**0.010**
Pregnancy loss	86(18.9)	21(30.0)	65(16.9)	
Gestational diabetes mellitus				
Yes	41(11.2)	10(20.4)	31(9.7)	**0.027**
No	327(88.8)	39(79.6)	288(90.3)	
Anaemia in pregnancy				
Yes	68(18.5)	12(24.5)	56(17.6)	0.244
No	300(81.5)	37(75.5)	263(82.4)	
Hypothyroidism in pregnancy				
Yes	64(13.7)	28(37.3)	36(9.2)	**<0.001**
No	402(86.3)	47(62.7)	355(90.8)	
Pregnancy-induced hypertension				
Yes	26(7.1)	6(12.2)	20(6.3)	0.136
No	342(92.9)	43(87.8)	299(93.7)	
Intrahepatic cholestasis of pregnancy				
Yes	10(2.7)	2(4.1)	8(2.5)	0.629
No	358(97.3)	47(95.9)	311(97.5)	
Fetal distress in uterus				
Yes	34(9.2)	6(12.2)	28(8.8)	0.428
No	334(90.8)	43(87.8)	291(91.2)	
Fetal growth restriction				
Yes	14(3.8)	4(8.2)	10(3.1)	0.101
No	354(96.2)	45(91.8)	309(96.9)	
Oligohydramnios				
Yes	30(8.2)	6(12.2)	24(7.5)	0.263
No	338(91.8)	43(87.8)	295(92.5)	
Premature rupture of membrane				
Yes	54(14.7)	13(26.5)	41(12.9)	**0.012**
No	314(85.3)	36(73.5)	278(87.1)	
Preterm birth				
Yes	27(7.3)	8(16.3)	19(6.0)	**0.017**
No	341(92.7)	41(83.7)	300(94.0)	
Postpartum hemorrhage				
Yes	22(6.0)	5(10.2)	17(5.3)	0.193
No	346(94.0)	44(89.8)	302(94.7)	

Data are presented as numbers and percentages. *P* values <0.05 were shown in bold.

**Figure 3:**
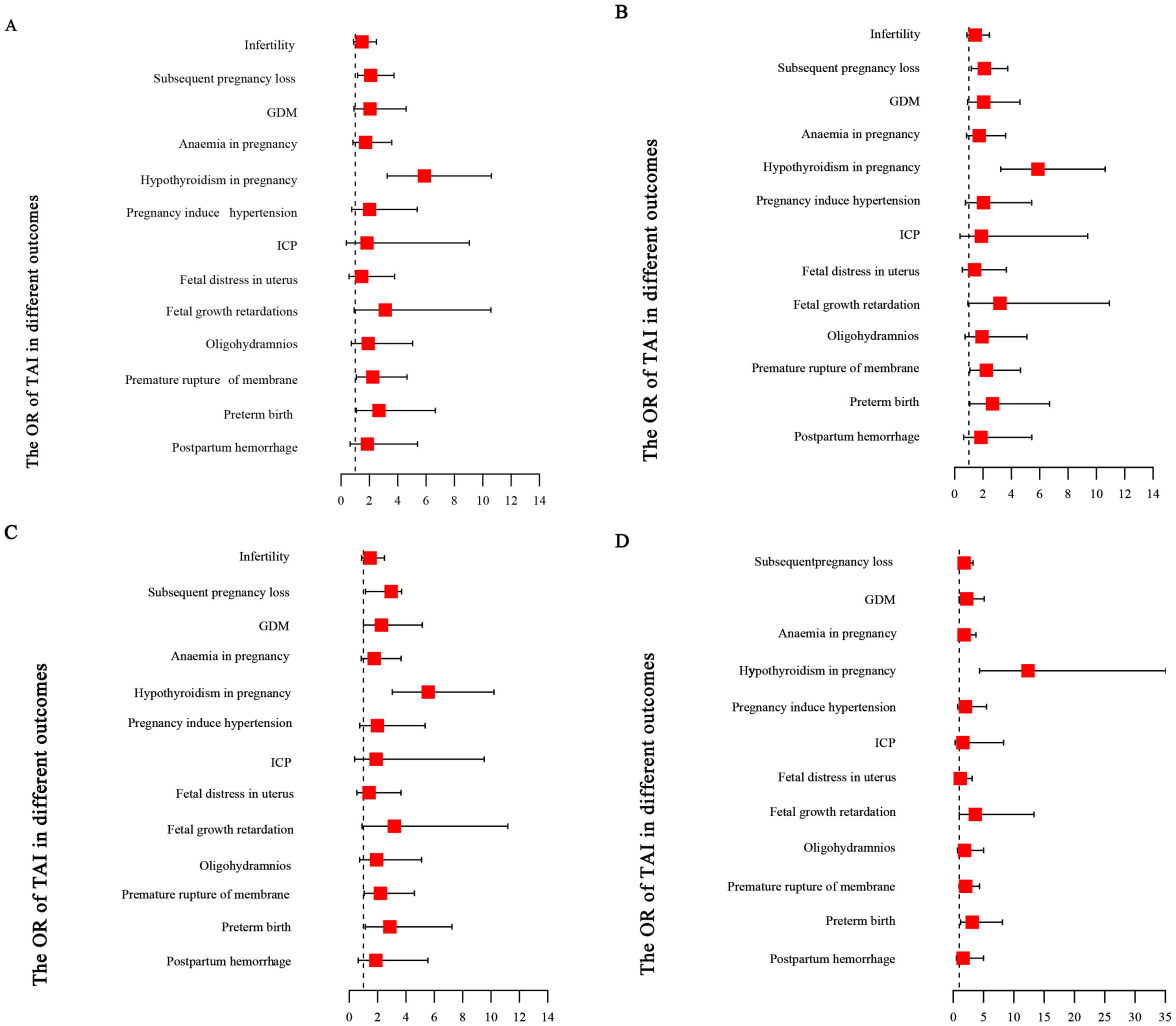
The forest plots of the associations among TAI and clinical outcomes and pregnancy complications. **(A)** Unadjusted; **(B)** Adjusted for age, BMI, total number of pregnancies, and number of pregnancy losses; **(C)** Additionally adjusted for pre-conception TSH and fT4; D: Further adjusted for first-trimester TSH and fT4.

Logistic regression analyses were performed to evaluate the association between TAI and clinical outcomes as well as pregnancy complications (**Table 4**, **Figure 3**). The presence of TAI was associated with increased odds of subsequent pregnancy loss (OR 2.103, 95% CI 1.182–3.744, P = 0.011), GDM (OR 2.382, 95% CI 1.084–5.235, P = 0.031), hypothyroidism during pregnancy (OR 5.876, 95% CI 3.289–10.493, P < 0.001), PROM (OR 2.449, 95% CI 1.199–5.000, P = 0.014), and preterm birth (OR 3.081, 95% CI 1.267–7.489, P = 0.013) (**Figure 3A**). After adjusting age, BMI, total number of pregnancies, number of pregnancy losses, and pre-pregnancy TSH and fT4, TAI remained positively associated with subsequent pregnancy loss (OR 2.953, 95% CI 1.142–3.693, P = 0.016), hypothyroidism during pregnancy (OR 5.567, 95% CI 3.035–10.210, P < 0.001), PROM (OR 2.198, 95% CI 1.051–4.595, P = 0.036), and preterm birth (OR 2.865, 95% CI 1.132–7.249, P = 0.026) (**Figure 3C**, **Table 4**). However, after further adjustment for first-trimester TSH and fT4, TAI was only significantly associated with hypothyroidism during pregnancy (OR 12.338, 95% CI 4.341–35.064, P < 0.001) and preterm birth (OR 3.138, 95% CI 1.213–8.117, P = 0.018) (**Figure 3D**, **Table 4**).

**Table 4:** Logistics regression of thyroid autoimmunity and infertility, pregnancy loss and pregnant complications.

	Model 1		Model 2		Model 3		Model 4	
	OR (95% CI)	*P*	OR (95% CI)	*P*	OR (95% CI)	*P*	OR (95% CI)	*P*
Infertility	1.482 (0.880-2.495)	0.139	1.452 (0.861-2.447)	0.162	1.469 (0.869-2.483)	0.151		
Subsequent pregnancy loss	**2.087 (1.166-3.734)**	**0.013**	**2.090 (1.167-3.744)**	**0.013**	**2.953 (1.142-3.693)**	**0.016**	1.790 (0.978-3.277)	0.059
GDM	2.043 (0.909-4.592)	0.084	2.048 (0.910-4.607)	0.083	2.270 (1.001-5.149)	0.050	2.226 (0.970-5.109)	0.059^a^
Anaemia in pregnancy	1.726 (0.833-3.576)	0.142	1.736 (0.837-3.602)	0.138	1.757 (0.844-3.655)	0.132	1.788 (0.852-3.751)	0.124^b^
Hypothyroidism in pregnancy	**5.876 (3.254-10.609)**	**<0.001**	**5.876 (3.252-10.619)**	**<0.001**	**5.567 (3.035-10.210)**	**<0.001**	**12.338 (4.341-35.064)**	**<0.001**
Pregnancy induce hypertension	2.011 (0.753-5.370)	0.163	2.031 (0.759-5.438)	0.158	1.989 (0.739-5.352)	0.173	2.018 (0.741-5.496)	0.170
ICP	1.830 (0.370-9.048)	0.458	1.883 (0.378-9.388)	0.440	1.895 (0.377-9.520)	0.437	1.595 (0.306-8.314)	0.580
Fetal distress in uterus	1.460 (0.564-3.778)	0.435	1.404 (0.541-3.647)	0.485	1.396 (0.535-3.646)	0.495	1.151 (0.426-3.115)	0.781^c^
Fetal growth restriction	3.118 (0.920-10.575)	0.068	3.192 (0.934-10.913)	0.064	3.177 (0.902-11.189)	0.072	3.650 (0.999-13.335)	0.050
Oligohydramnios	1.914 (0.725-5.050)	0.190	1.929 (0.729-5.103)	0.186	1.924 (0.726-5.098)	0.188	1.859 (0.689-5.021)	0.221
Premature rupture of membrane	**2.238 (1.077-4.655)**	**0.031**	**2.231 (1.072-4.646)**	**0.032**	**2.198 (1.051-4.595)**	**0.036**	2.045 (0.964-4.338)	0.062^d^
Preterm birth	**2.674 (1.075-6.652)**	**0.034**	**2.669 (1.064-6.692)**	**0.036**	**2.865 (1.132-7.249)**	**0.026**	**3.138 (1.213-8.117)**	**0.018**
Postpartum hemorrhage	1.858 (0.640-5.396)	0.255	1.854 (0.632-5.443)	0.261	1.872 (0.632-5.546)	0.258	1.633 (0.534-5.000)	0.390^e^

GDM: gestational diabetes mellitus; ICP: intrahepatic cholestasis of pregnancy.

Model 1: Adjusted for age and BMI; Model 2: Model 1+ total pregnancy numbers and PL numbers; Model 3: Model 2+ TSH and fT4 in pre-pregnancy; Model 4: Model 3+TSH and fT4 in first trimester.

a The association between TAI and GDM were further adjusted by the confounders in Model 4 and *fasting blood glucose and* 2 hours postprandial blood glucose in pre-pregnancy, and the OR is 2.309(95% CI 0.981-5.439), *P* = 0.056.

b The association between TAI and anaemia in pregnancy were further adjusted by the confounders in Model 4 and Hb in pre-pregnancy, and the OR is 1.709(95% CI 0.807-3.619), *P* = 0.161.

c The association between TAI and fetal growth restriction were further adjusted by the confounders in Model 4 and race and Hb in pre-pregnancy, and the OR is 3.261(95% CI 0.874-12.170), *P* = 0.079.

d The association between TAI and premature rupture of membrane were further adjusted by the confounders in Model 4 and WBC and *neutrophils* in pre-pregnancy, and the OR is 1.955(95% CI 0.909-4.204), *P* = 0.086.

e The association between TAI and postpartum hemorrhage were further adjusted by the confounders in Model 4 and delivery method and foetal weight, and the OR is 1.606(95% CI 0.518-4.982), *P* = 0.412.

*P* values <0.05 were shown in bold.

## 4 Discussion

The prevalence of TAI in women of reproductive age is 8–14%, but rises in women with RPL (17, 29). Previous studies have linked TAI to increased risks of sporadic miscarriage, preterm delivery and subfertility (17, 18, 29). However, the relationship between TAI and URPL remains unclear. Testing for TPO-Ab and TG-Ab may help predict clinical outcomes in women with URPL. To date, most evidence relating TAI to fertility and pregnancy outcomes has come from retrospective studies. We therefore conducted a prospective cohort study of euthyroid women with URPL to examine this question. Our findings indicated that infertility rates did not differ significantly between women with and without TAI. Pregnancy complications tended to be more common in women with TAI. The logistic regression analysis showed that TAI was positively associated with GDM, hypothyroidism in pregnancy, PROM, and preterm birth.

Although TAI has been linked to reproductive challenges (17, 18), its impact on clinical outcomes in euthyroid women with URPL remains unknown. Proposed mechanisms include subtle thyroid dysfunction and a broader breakdown of immunotolerance (17). Therefore, we hypothesized that TAI may serve as a marker of immune dysfunction in women with RPL. In this study, we investigated whether TAI could predict clinical outcomes in women with URPL.

The levels of thyroid hormones during pregnancy are influenced by various factors, such as age and BMI (41). In our study, participants in the TAI group had a higher BMI, were older at the time of their first pregnancy, were more likely to belong to other ethnic backgrounds, and were more likely to possess a bachelor’s degree or above compared to those in the non-TAI group. These findings are consistent with previous studies. For example, Derakhshan et al. indicated that BMI and gestational age are the main determinants of TSH (BMI only), fT4, and fT3 (42). Similarly, Lin et al. observed that a BMI ≥ 23 kg/m^2^, maternal parity ≥3, and maternal age ≥30 years were associated with an increased risk of thyroid dysfunction (43). Cárdenas Roldán J. also observed a positive association between higher educational attainment and autoimmune thyroid disease (44). Moreover, the degree of TSH decline during pregnancy varies considerably across racial and ethnic groups. For instance, preliminary studies in pregnant women from the United States and Europe suggested an upper reference limit for TSH of 2.5 mU/L in early pregnancy, whereas for Chinese pregnant women, it is 4.34 mU/L (33).

While tracking thyroid markers from pre-pregnancy to the first trimester, we observed an interesting phenomenon: in the TAI group, first-trimester TSH levels remained unchanged from pre-pregnancy values, whereas a significant decrease was noted in the non-TAI group. The lack of a significant decrease in TSH in the TAI group during pregnancy appears to contrast with current understanding. Previous studies have shown that, due to the high affinity of HCG for TSH receptors, the rapid increase in HCG during early pregnancy stimulates the release of serum free thyroxine, which then leads to a decreased TSH concentrations via negative feedback compared to the non-pregnant state (43). However, in our study of euthyroid URPL women, first-trimester TSH levels in the TAI group did not decrease. Similarly, Glinoer et al. found slightly higher first-trimester TSH in TAI-positive euthyroid women and documented their progression toward hypothyroidism despite falling antibody titers (45). We therefore postulate that TAI attenuates thyroidal response to HCG during pregnancy, suggesting the need for closer surveillance even in women with normal pre-conception thyroid function (46).

Several studies have demonstrated that women with TAI are at an increased risk for obstetric complications (17, 19, 29). Consistent with these findings, our study observed that TAI was associated with a higher risk of subsequent pregnancy loss, hypothyroidism in pregnancy, PROM, and preterm birth. However, the relationship between TPO-Ab positivity and live birth rate (LBR) in URPL women remains controversial (47, 48). For example, Rushworth et al. and Meilan Liu et al. reported that no significant difference in LBR between URPL patients with or without TPO-Ab positivity (25, 49). In contrast, Bliddal’s study involving 825 women with URPL found that TPO-Ab positivity may predict a decreased LBR, and that LT4 treatment improved the likelihood of live birth (50). Other studies, however, have reported that LT4 treatment does not impact LBR (51). A multicenter, randomized, double-blind, placebo-controlled, phase 3 trial (T4LIFE trial) indicated that LT4 treatment failed to increase LBR in TPO-Ab-positive women with normal thyroid function and a history of RPL, or any secondary outcomes, including pregnancy losses, ongoing pregnancy rates, and preterm birth (31). Consistently, our research also found that even when LT4 was used promptly, TAI was still associated with adverse pregnancy outcomes. TAI may link to pregnancy complications via subclinical thyroid dysfunction, direct interference with placentation, or broader maternal-fetal immune imbalance (49). Consequently, LT4 alone may not improve pregnancy outcomes, and the underlying pathway remains to be clarified. Based on current evidence, we do not advise routine use of LT4 in URPL women with TAI and normal thyroid function. Furthermore, previous studies have shown that euthyroid RPL patients with positive TPO-Ab are at increased risk for early pregnancy loss (52). Consequently, our study demonstrated that most pregnancy losses occurred in the first trimester, and women with TAI had a higher risk of pregnancy loss compared to those without TAI, in line with previous meta-analyses (47, 53). TAI may induce early miscarriage by triggering the immune system and hindering the development of immunological tolerance, even though the precise mechanism is unknown yet (54). Therefore, greater attention should be given to URPL women with TAI.

Infertility affects a growing number of women of reproductive age. In our study, the infertility rate was 23.6% in the TAI group and 17.7% in the non-TAI group; however, this difference was not statistically significant. These findings are consistent with recent large-scale randomized controlled trials and meta-analyses (52, 55). Regarding the relationship between TAI and infertility in euthyroid women, three main theories have been proposed (56). First, TAI may not be pathogenic itself, but instead reflects generalized autoimmunity (57). Second, thyroid antibodies may exert a direct pathogenic effect on ovarian tissue (58). Third, TAI may cause a state of relative hypothyroidism that negatively affects reproductive tissues (59). The effects of TAI on fertility remain unclear and warrant further investigation.

Mechanistically, the association between TAI and reproductive health may be either direct or indirect. Specifically, TPO-Ab and Tg-Ab can cross the blood-follicle barrier and accumulate in follicular fluid at concentrations comparable to those in serum (60). Within the follicle, these antibodies can trigger antibody-dependent cell-mediated cytotoxicity and activate the C3-complement pathway, thereby compromising oocyte maturation, fertilization, and early embryo cleavage (61). This mechanistic link is supported by the consistently lower success rates of *in-vitro* fertilization in women who test positive for TAI (60). In addition, TAI may lead to immunological dysfunction and impaired immune tolerance, affecting both systemic immunity and the maternal-fetal interface. Women with TAI have been found to exhibit a systemic imbalance among helper T lymphocytes (Th1, Th2, Th17) and regulatory T cells (Tregs), resulting in increased secretion of inflammatory cytokines such as interleukin (IL)-2, IL-17, and interferon-γ (IFN-γ), all of which are associated with implantation failure and pregnancy loss (62, 63). Furthermore, studies have documented heightened activation and cytotoxic effects of natural killer (NK) cells in both the peripheral circulation and uterine environment, along with increased expression of NKT-like cells (56, 64, 65). Notably, NK cell quantity and functional activity may be stimulated not only by IL-2 and INF-γ, but also by TSH via endocrine and paracrine pathways.

This study has certain clinical implications. Our findings highlight the importance of pre-pregnancy TAI screening in women with URPL, even in the absence of thyroid dysfunction. The independent association of TAI with increased risks of subsequent pregnancy loss, hypothyroidism in pregnancy, PROM, and preterm birth suggests that clinicians should assess thyroid autoantibodies in women with URPL. For those identified as TAI-positive, several clinical measures are recommended: preconception counseling to inform patients of potential risks; close monitoring of thyroid function (TSH and fT4) before and during pregnancy, with more frequent testing in early gestation; and heightened surveillance for pregnancy complications, including PROM and preterm labor. Coordination between obstetricians and endocrinologists may further support optimal outcomes for both mother and fetus. Implementing these strategies may facilitate early detection and intervention, potentially reducing adverse pregnancy outcomes. Future large-scale studies are needed to refine risk stratification and develop standardized management protocols for TAI in the context of URPL.

Strengths of this study include strict inclusion-exclusion criteria and complete data on thyroid function. However, several limitations should be acknowledged. First, the modest sample size may have rendered the study underpowered to detect subtle associations. Second, as participants were not randomly assigned to groups, the study may have been subject to allocation and selection biases. Third, insufficient treatment data precluded evaluation of the potential effects of interventions, and heterogeneity in treatment strategies during pregnancy may have further confounded the outcomes. Fourth, single-center recruitment and the predominantly Han-Chinese composition limit the generalisability of our findings to broader or ethnically diverse populations. To validate our findings, more prospective randomized, polycentric, and large population investigations are required.

## Data Availability

The original contributions presented in the study are included in the article/**Supplementary Material**. Further inquiries can be directed to the corresponding author.
