# Correction: Association of thyroid autoimmunity and pregnancy outcomes in unexplained recurrent pregnancy loss women: a prospective cohort study

**DOI:** 10.3389/fendo.2025.1756216

**Published:** 2025-12-19

**Authors:** Ruifang Wang, Ling Liu, Wei Zhang, Jian Zhang, Kexin Wang, Fang Wang

**Affiliations:** 1 Department of Reproductive Medicine, Lanzhou University Second Hospital, Lanzhou, China; 2 Department of Obstetrics and Gynecology, The First Affiliated Hospital, and College of Clinical Medicine of Henan University of Science and Technology, Luoyang, China

**Keywords:** thyroid autoimmunity, unexplained recurrent pregnancy loss, fertility, subsequent pregnancy outcome, pregnancy complications

In the published article, there was an error in **Table 1** as published. Under the “Variable” column for “TSH” in **Table 1**, the first category, <2.5, was inadvertently omitted. The corrected **Table 1** and its caption appear below.

**Table 1:** Baseline characteristics of 576 euthyroid women with URPL.

Variables	Whole patients (N=576)	TAI group (N=101)	Non-TAI group (N=475)	*P*
Maternal Age (years)	30.3±4.0	30.9±3.7	30.2±4.1	0.110
BMI (kg/m^2^)	22.4±3.1	23.0±3.5	22.2±3.0	**0.017**
BMI (kg/m^2^)				
<18.5	47(8.2)	6(5.9)	41(8.6)	0.080
18.5-23.9	393(68.2)	63(62.4)	330(69.5)	
24-27.9	101(17.5)	21(20.8)	80(16.8)	
≥28(obesity)	35(6.1)	11(10.9)	24(5.1)	
Ethic				
Han	515(89.4)	84(83.2)	431(90.7)	**0.025**
Other	61(10.6)	17(16.8)	44(9.3)	
Education				
Junior middle school and below	102(17.7)	10(9.9)	92(19.4)	**0.017**
High school and jnior college	241(41.8)	39(38.6)	202(42.5)	
Bachelor’s degree or above	233(40.5)	52(51.5)	181(38.1)	
Age at menarche (years)	13.5±1.2	13.6±1.3	13.4±1.2	0.130
Age at first pregnancy (years)	26.0±3.4	26.6±3.4	25.9±3.4	**0.038**
Number of total pregnancy	2.9±1.1	2.9±0.9	2.9±1.1	0.976
Number of pregnancy loss	2.5±0.8	2.4±0.8	2.5±0.8	0.385
Type of pregnancy loss				
Primary	446(77.4)	72(71.3)	374(78.7)	0.104
Secondary	130(22.6)	29(28.7)	101(21.3)	
Thyroid function before pregnancy				
T3 (nmol/L)	1.8±0.3	1.9±0.3	1.8±0.3	0.237
T4 (nmol/L)	111.2±19.6	112.7±19.1	110.8±19.7	0.375
fT3 (pmol/L)	5.2±0.5	5.1±0.4	5.2±0.5	**0.018**
fT4 (ng/ml)	15.9±1.9	15.9±1.9	15.9±1.9	0.810
TSH (mIU/L)	2.2±0.9	2.4±0.9	2.2±0.8	**0.037**
TSH (mIU/L)				
<2.5	368(63.9)	59(58.4)	309(65.1)	0.207
≥ 2.5 to ≤ 4.2	208(36.1)	42(41.6)	166(34.9)	

Continuous variables are described as mean and standard deviation, categorical variables are expressed as numbers and percentages. BMI, body mass index; T3, triiodothyronine; T4, thyroxine; fT3, free triiodothyronine; fT4, free thyroxine; TSH, thyroid stimulating hormone.

P values <0.05 were shown in bold.

The original article has been updated.

