# Supplementary material for: Association of thyroid autoimmunity and pregnancy outcomes in unexplained recurrent pregnancy loss women: a prospective cohort study
Source: Front Endocrinol (Lausanne). 2025 Nov 25;16:1711369. doi: 10.3389/fendo.2025.1711369 (PMC12685602; doi:10.3389/fendo.2025.1711369)
Supplement: Supplementary file 1 [file Table1.doc]

**Supplement Table 1 Baseline characteristics and thyroid function parameters of all subsequent pregnant patients.**

| **Variables** | **Whole patients (N=466)** | **TAI group**  **(N=75)** | **Non-TAI group**  **(N=391)** | ***P*** |
| --- | --- | --- | --- | --- |
| **Maternal Age (years)** | 29.9±3.8 | 30.4±3.4 | 29.7±3.9 | 0.152 |
| **BMI(kg/m^2^)** | 22.2±2.9 | 23.1±3.4 | 22.1±2.8 | **0.019** |
| **BMI(kg/m^2^)** |  |  |  | **0.009** |
| <18.5 | 39(8.4) | 5(6.7) | 34(8.7) |
| 18.5-23.9 | 321(68.9) | 45(60.0) | 276(70.6) |
| 24-27.9 | 82(17.6) | 15(20.0) | 67(17.1) |
| ≥28(obesity) | 24(5.2) | 10(13.3) | 14(3.6) |
| **Ethic** |  |  |  | **0.042** |
| Han | 421(90.3) | 63(84.0) | 358(91.6) |
| Other | 45(9.7) | 12(16.0) | 33(8.4) |
| **Education** |  |  |  | 0.212 |
| Junior middle school and below | 87(18.7) | 10(13.3) | 77(19.7) |
| High school and junior college | 194(41.6) | 29(38.7) | 165(42.3) |
| Bachelor degree and above | 185(39.7) | 36(48.0) | 149(38.1) |
| **Age at menarche (years)** | 13.4±1.2 | 13.6±1.3 | 13.4±1.2 | 0.160 |
| **Age at first pregnancy (years)** | 25.9±3.5 | 26.3±3.3 | 25.8±3.5 | 0.278 |
| **Number of total pregnancy** | 2.9±1.1 | 2.9±1.0 | 2.9±1.1 | 0.559 |
| **Number of pregnancy loss** | 2.5±0.8 | 2.5±0.9 | 2.5±0.8 | 0.835 |
| **Type of pregnancy loss** |  |  |  | 0.106 |
| Primary | 368(79.0) | 54(72.0) | 314(80.3) |
| Secondary | 98(21.0) | 21(28.0) | 77(19.7) |
| **Thyroid function before pregnancy** |  |  |  |  |
| T3(nmol/L) | 1.8±0.3 | 1.8±0.3 | 1.8±0.3 | 0.778 |
| T4(nmol/L) | 111.6±19.7 | 112.8±19.3 | 111.4±19.8 | 0.571 |
| fT3(pmol/L) | 5.2±0.5 | 5.1±0.4 | 5.3±0.5 | **0.038** |
| fT4(ng/ml) | 15.9±1.9 | 15.7±1.9 | 16.0±1.9 | 0.227 |
| TSH(mIU/L) | 2.2±0.9 | 2.3±0.8 | 2.2±0.9 | 0.085 |
| **TSH(mIU/L)** |  |  |  | 0.341 |
| <2.5 | 296(63.5) | 44(58.7) | 252(64.5) |
| ≥ 2.5 to ≤ 4.2 | 170(36.5) | 31(41.3) | 139(35.5) |
| **Thyroid function in first trimester** |  |  |  |  |
| T3(nmol/L) | 1.8±0.3 | 1.7±0.4 | 1.8±0.3 | **0.021** |
| T4(nmol/L) | 106.5±18.7 | 104.6±23.1 | 106.8±17.8 | 0.437 |
| fT3(pmol/L) | 5.0±0.5 | 4.7±0.7 | 5.0±0.5 | **<0.001** |
| fT4(ng/ml) | 14.8±2.1 | 14.2±2.8 | 14.9±1.9 | **0.024** |
| TSH(mIU/L) | 1.8±0.9 | 2.1±1.2 | 1.7±0.9 | **0.014** |
| **TSH(mIU/L)** |  |  |  | **0.001** |
| <2.5 | 397(85.2) | 55(73.3) | 342(87.5) |
| ≥ 2.5 to ≤ 4.0 | 56(12.0) | 14(18.7) | 42(10.7) |
| >4.0 | 13(2.8) | 6(8.0) | 7(1.8) |

Continuous variables are described as mean and standard deviation, categorical variables are expressed as numbers and percentages. BMI, body mass index; T3, triiodothyronine; T4, thyroxine; fT3, free triiodothyronine; fT4, free thyroxine; TSH, thyroid stimulating hormone.

*P* values <0.05 were shown in bold.

**Supplement Table 2 Baseline characteristics and thyroid function parameters in pregnant patients with live birth and pregnancy loss.**

| **Variables** | **Whole patients (N=454)** | **TAI group**  **(N=70)** | **Non-TAI group**  **(N=384)** | ***P*** |
| --- | --- | --- | --- | --- |
| **Maternal Age (years)** | 29.8±3.9 | 30.4±3.5 | 29.7±3.9 | 0.175 |
| **BMI(kg/m^2^)** | 22.2±2.8 | 23.0±3.4 | 22.1±2.7 | **0.040** |
| **BMI(kg/m^2^)** |  |  |  | **0.015** |
| <18.5 | 38(8.4) | 5(7.1) | 33(8.6) |
| 18.5-23.9 | 316(69.6) | 43(61.4) | 273(71.1) |
| 24-27.9 | 78(17.2) | 13(18.6) | 65(16.9) |
| ≥28(obesity) | 22(4.8) | 9(12.9) | 13(3.4) |
| **Ethic** |  |  |  | **0.028** |
| Han | 409(90.1) | 58(82.9) | 351(91.4) |
| Other | 45(9.9) | 12(17.1) | 33(8.6) |
| **Education** |  |  |  | 0.176 |
| Junior middle school and below | 84(18.5) | 10(14.3) | 74(19.3) |
| High school and junior college | 188(41.4) | 25(35.7) | 163(42.4) |
| Bachelor degree and above | 182(40.1) | 35(50.0) | 147(38.3) |
| **Age at menarche (years)** | 13.4±1.2 | 13.6±1.3 | 13.4±1.2 | 0.148 |
| **Age at first pregnancy (years)** | 25.8±3.5 | 26.2±3.4 | 25.8±3.5 | 0.358 |
| **Number of total pregnancy** | 2.9±1.1 | 2.9±1.0 | 2.9±1.1 | 0.713 |
| **Number of pregnancy loss** | 2.5±0.8 | 2.4±0.9 | 2.5±0.8 | 0.617 |
| **Type of pregnancy loss** |  |  |  | 0.164 |
| Primary | 359(79.1) | 51(72.9) | 308(80.2) |
| Secondary | 95(20.9) | 19(27.1) | 76(19.8) |
| **Thyroid function before pregnancy** |  |  |  |  |
| T3(nmol/L) | 1.8±0.3 | 1.8±0.3 | 1.8±0.3 | 0.593 |
| T4(nmol/L) | 111.5±19.6 | 112.7±18.5 | 111.3±19.8 | 0.590 |
| fT3(pmol/L) | 5.2±0.5 | 5.1±0.5 | 5.3±0.5 | 0.054 |
| fT4(ng/ml) | 15.9±1.9 | 15.7±1.9 | 16.0±1.9 | 0.213 |
| TSH(mIU/L) | 2.2±0.9 | 2.4±0.8 | 2.2±0.9 | 0.062 |
| **TSH(mIU/L)** |  |  |  | 0.252 |
| <2.5 | 287(63.2) | 40(57.1) | 247(64.3) |
| ≥ 2.5 to ≤ 4.2 | 167(36.8) | 30(42.9) | 137(35.7) |
| **Thyroid function in first trimester** |  |  |  |  |
| T3(nmol/L) | 1.8±0.3 | 1.7±0.4 | 1.8±0.3 | 0.089 |
| T4(nmol/L) | 106.6±18.7 | 104.2±23.0 | 106.8±17.9 | 0.582 |
| fT3(pmol/L) | 5.0±0.5 | 4.6±0.7 | 5.0±0.5 | **<0.001** |
| fT4(ng/ml) | 14.8±2.1 | 14.3±2.8 | 14.9±1.9 | 0.070 |
| TSH(mIU/L) | 1.8±0.9 | 2.1±1.1 | 1.7±0.9 | **0.014** |
| **TSH(mIU/L)*** |  |  |  | **0.007** |
| <2.5 | 387(85.2) | 52(74.3) | 335(87.2) |
| ≥ 2.5 to ≤ 4.0 | 55(12.1) | 13(18.6) | 42(10.9) |
| >4.0 | 12(2.6) | 5(7.1) | 7(1.8) |

Continuous variables are described as mean and standard deviation, categorical variables are expressed as numbers and percentages. BMI, body mass index; T3, triiodothyronine; T4, thyroxine; fT3, free triiodothyronine; fT4, free thyroxine; TSH, thyroid stimulating hormone.

*P* values <0.05 were shown in bold.

**Supplement Table 3 Comparisons of thyroid function parameters between pre-pregnancy and first trimester in pregnant patients with live birth and pregnancy loss.**

|  | **TAI group(N=70)**  **N=75** | | ***P_1_*** | **Non-TAI group(N=384)**  **N=** | | ***P_2_*** |
| --- | --- | --- | --- | --- | --- | --- |
|  | **Pre-pregnancy** | **First-trimester** |  | **Pre-pregnancy** | **First-trimester** |  |
| **T3(nmol/L)** | 1.8±0.3 | 1.7±0.4 | **0.038** | 1.8±0.3 | 1.8±0.3 | 0.153 |
| **T4(nmol/L)** | 112.7±18.5 | 105.2±23.0 | **0.037** | 111.3±19.8 | 106.8±17.9 | **0.001** |
| **fT3(pmol/L)** | 5.1±0.5 | 4.6±0.7 | **<0.001** | 5.3±0.5 | 5.0±0.5 | **<0.001** |
| **fT4(ng/ml)** | 15.7±1.9 | 14.3±2.8 | **<0.001** | 16.0±1.9 | 14.9±1.9 | **<0.001** |
| **TSH(mIU/L)** | 2.4±0.8 | 2.1±1.1 | 0.094 | 2.2±0.9 | 1.7±0.9 | **<0.001** |
| **TPO-Ab(mIU/L)** | 319.7±452.7 | 311.7±431.3 | 0.915 | 28.6±15.4 | 28.9±18.6 | 0.780 |
| **TG-Ab(mIU/L)** | 114.2±120.3 | 120.2±102.9 | 0.753 | 18.1±10.2 | 17.5±13.7 | 0.478 |
| **TSH(mIU/L)** |  |  | **0.012** |  |  | **<0.001** |
| <2.5 | 40(57.1) | 52(74.3) |  | 247(64.3) | 335(87.2) |  |
| ≥ 2.5 to ≤ 4.0 | 28(40.0) | 13(18.6) |  | 132(34.4) | 42(10.9) |  |
| >4.0 | 2(2.9) | 5(7.1) |  | 5(1.3) | 7(1.8) |  |

Continuous variables are described as mean and standard deviation, categorical variables are expressed as numbers and percentages. T3, triiodothyronine; T4, thyroxine; fT3, free triiodothyronine; fT4, free thyroxine; TSH, thyroid stimulating hormone; TPO-Ab, thyroid peroxidase antibody; TG-Ab, antithyroglobulin antibody.

*P* values <0.05 were shown in bold.

*P_1_,* pre-pregnancy data vs first first-trimester data in TAI group; *P_2_,* pre-pregnancy data vs. first first-trimester data in non-TAI group.

**Supplement Table 4 Comparisons of thyroid function parameters in pre-pregnancy and first trimester in pregnant patients who had live birth or pregnancy.**

|  | **Live birth group(N=368)**  **N=75** | | ***P_1_*** | **Pregnancy loss group(N=86)**  **N=** | | ***P_2_*** |
| --- | --- | --- | --- | --- | --- | --- |
|  | **Pre-pregnancy** | **First-trimester** |  | **Pre-pregnancy** | **First-trimester** |  |
| **T3(nmol/L)** | 1.8±0.3 | 1.8±0.3 | **0.013** | 1.8±0.3 | 1.8±0.4 | 0.970 |
| **T4(nmol/L)** | 112.1±19.9 | 107.0±18.7 | **<0.001** | 109.1±18.4 | 104.9±19.0 | 0.143 |
| **fT3(pmol/L)** | 5.2±0.5 | 5.0±0.5 | **<0.001** | 5.2±0.5 | 5.0±0.6 | **0.015** |
| **fT4(ng/ml)** | 15.9±1.9 | 15.0±1.9 | **<0.001** | 15.9±1.9 | 14.3±2.7 | **<0.001** |
| **TSH(mIU/L)** | 2.2±0.9 | 1.7±0.9 | **<0.001** | 2.3±0.8 | 2.0±1.1 | 0.084 |
| **TPO-Ab(mIU/L)** | 72.5±207.4 | 73.5±206.2 | 0.950 | 77.4±201.8 | 68.4±156.8 | 0.743 |
| **TG-Ab(mIU/L)** | 30.1±55.2 | 31.0±51.1 | 0.814 | 45.0±72.7 | 43.2±73.5 | 0.868 |
| **TSH(mIU/L)** |  |  | **<0.001** |  |  | **<0.001** |
| <2.5 | 235(63.9) | 320(87.0) |  | 52(60.5) | 67(77.9) |  |
| ≥ 2.5 to ≤ 4.0 | 133(36.1) | 43(11.7) |  | 34(39.5) | 12(26.1) |  |
| >4.0 | 0(0.0) | 5(1.4) |  | 0(0.0) | 7(8.1) |  |

Continuous variables are described as mean and standard deviation, categorical variables are expressed as numbers and percentages. T3, triiodothyronine; T4, thyroxine; fT3, free triiodothyronine; fT4, free thyroxine; TSH, thyroid stimulating hormone; TPO-Ab, thyroid peroxidase antibody; TG-Ab, antithyroglobulin antibody.

*P* values <0.05 were shown in bold.

*P_1_,* pre-pregnancy data vs first first-trimester data in TAI group; *P_2_,* pre-pregnancy data vs. first first-trimester data in non-TAI group.
